# The posterior tibial slope modifies the diagnostic utility of posterior shiny‐corner lesions in medial meniscus posterior root tears

**DOI:** 10.1002/jeo2.70843

**Published:** 2026-07-09

**Authors:** Ryo Sasaki, Taichi Nishimura, Teppei Hayashi, Kazuya Kaneda, Masaki Nagashima, Hideo Morioka

**Affiliations:** ^1^ Department of Orthopaedic Surgery NHO Tokyo Medical Center Meguro‐ku Tokyo Japan; ^2^ Department of Orthopaedic Surgery Keio University School of Medicine Shinjuku‐ku Tokyo Japan; ^3^ Department of Orthopaedic Surgery International University of Health and Welfare Mita Hospital Minato‐ku, Tokyo Japan

**Keywords:** diagnosis, magnetic resonance imaging, medial meniscus posterior root tear, posterior shiny‐corner lesion, posterior tibial slope

## Abstract

**Purpose:**

A posterior shiny‐corner lesion (PSCL) on magnetic resonance imaging (MRI) is an early imaging marker of medial meniscus posterior root tear (MMPRT). However, false‐negative PSCL findings may occur even in the acute phase of MMPRT. This study aimed to investigate whether the posterior tibial slope (PTS) modifies the presence of PSCL in surgically treated MMPRT across different MRI timing categories. We hypothesized that higher PTS would be associated with PSCL positivity.

**Methods:**

We retrospectively reviewed records of 53 knees in 46 patients (36 women and 10 men; mean age, 66.5 ± 9.4 years) treated surgically for MMPRT who had undergone preoperative MRI. MRI timing from symptom onset was categorized as acute (within 3 weeks), subacute (within 8 weeks but beyond 3 weeks), and chronic (beyond 8 weeks). The presence of PSCL, MRI signs (cleft, giraffe neck and ghost signs), and medial meniscus extrusion were recorded. Multivariable logistic regression analysis was performed to evaluate whether PTS was independently associated with PSCL after adjusting for MRI timing category.

**Results:**

The prevalence of PSCL decreased with time from onset (acute, 86.4%, 19/22 knees; subacute, 62.5%, 10/16 knees; chronic, 13.3%, 2/15 knees). PSCL‐positive patients had higher PTS than PSCL‐negative patients (8.7° ± 3.4° vs. 5.5° ± 2.9°; *p* < 0.001). In the multivariable logistic regression adjusted for time category, a higher PTS was independently associated with PSCL positivity (odds ratio, 1.35 per 1°; 95% confidence interval, 1.05–1.74; *p* = 0.021).

**Conclusion:**

PSCL is associated with early phase of MMPRT; however, its presence is influenced by PTS and is not solely time‐dependent. PSCL may be absent even in acute cases with low PTS and therefore should not be used alone to exclude acute MMPRT.

**Level of Evidence:**

Level III.

**Abbreviations**
CI: confidence interval
ICC: intraclass correlation coefficient
MME: medial meniscus extrusion
MMPRT: medial meniscus posterior root tear
MRI: magnetic resonance imaging
OR: odds ratio
PSCL: posterior shiny‐corner lesion
PTS: posterior tibial slope
SIFK: subchondral insufficiency fracture of the knee
SONK: spontaneous osteonecrosis of the knee

## INTRODUCTION

Magnetic resonance imaging (MRI) is the main diagnostic imaging modality for medial meniscus posterior root tear (MMPRT), and several imaging signs have been described [5]. Among them, a posterior shiny‐corner lesion (PSCL) at the posterior tibial corner on fluid‐sensitive sequences has been proposed as a sensitive early marker of MMPRT, as it is thought to reflect a focal bone marrow reaction related to acute impaction at the meniscal root attachment. Accordingly, PSCL has been suggested as a potential indicator of recent‐onset MMPRT [13].

However, in clinical practice, the timing of symptom onset is often unclear, and determining whether a lesion is acute or chronic is essential for treatment decision‐making, particularly when considering root repair [4, 11]. Although PSCL is frequently interpreted as an early‐phase marker, its reliability as an indicator of acute MMPRT remains uncertain, especially in cases where PSCL is absent despite recent symptoms.

In contrast, medial meniscus extrusion (MME) has been reported to increase progressively over time and is often regarded as a marker of chronic disease [6, 15]. Therefore, clinicians are often required to interpret PSCL and MME together when estimating lesion chronicity; however, the factors influencing PSCL visibility have not been fully clarified.

The posterior tibial slope (PTS) is a known anatomic risk factor for MMPRT and is associated with increased posterior shear force and meniscal stress [1, 3, 10, 14]. As PSCL is thought to reflect a localized subchondral reaction, it is plausible that tibial morphology, particularly PTS, may influence PSCL appearance. This may lead to false‐negative PSCL findings even in acute MMPRT, thereby limiting its diagnostic utility. Therefore, the purpose of this study was to investigate whether PTS modifies the presence of PSCL across different MRI timing categories, with a focus on its implications for interpreting PSCL as a marker of lesion chronicity. We hypothesized that a higher PTS would be independently associated with PSCL positivity.

## METHODS

### Study design and participants

This retrospective cohort study included 46 consecutive patients (36 women and 10 men) who were treated surgically for MMPRT at a single institution between February 2019 and September 2025. Inclusion criteria were a diagnosis of MMPRT confirmed by preoperative MRI and intraoperative findings, availability of preoperative knee MRI, and surgical treatment at our institution. No additional exclusion criteria were applied. Intraoperatively, MMPRT was defined as a tear involving the medial meniscus posterior root attachment or a radial tear adjacent to the posterior root attachment that functionally disrupted the posterior root; this definition allowed inclusion of both complete root avulsions and radial tears close to the posterior root attachment.

### MRI timing category

The time from symptom onset or injury to MRI was categorized as acute (within 3 weeks), subacute (within 8 weeks but beyond 3 weeks) and chronic (beyond 8 weeks) [13].

### MRI evaluation

**Figure 1:**
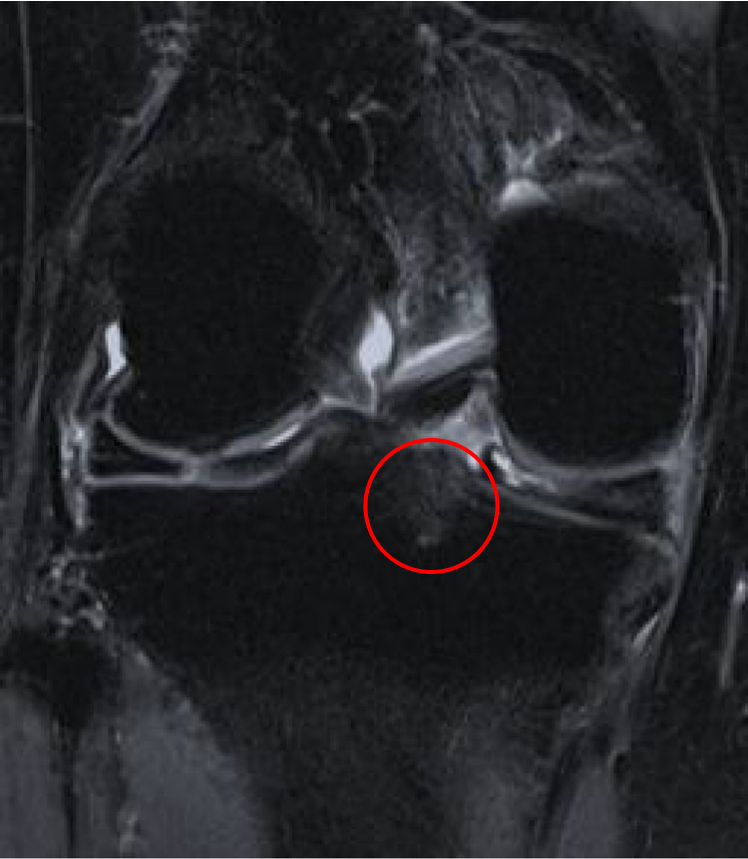
Magnetic resonance image of a posterior shiny‐corner lesion. Red circle, posterior shiny‐corner lesion.

**Figure 2:**
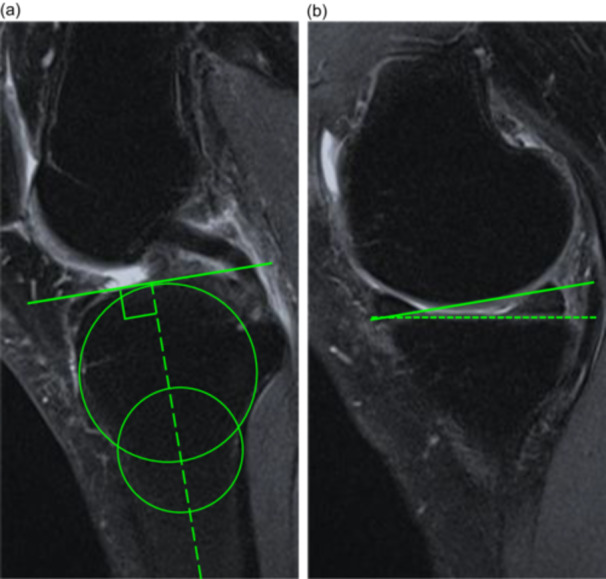
Magnetic resonance image of the posterior tibial slope. (a) On the sagittal plane in which the centre of the posterior cruciate ligament is visualized, two circles are drawn, each tangent to the cortical margins of the bone. The line connecting the centres of these circles is defined as the tibial axis (long dashed line). The solid line is perpendicular to the tibial axis. (b) On the sagittal plane in which the posterior slope of the medial tibial plateau is visualized, the posterior tibial slope is measured as the angle between the solid line and the dashed line connecting the most superior anterior and posterior cortical edges.

**Figure 3:**
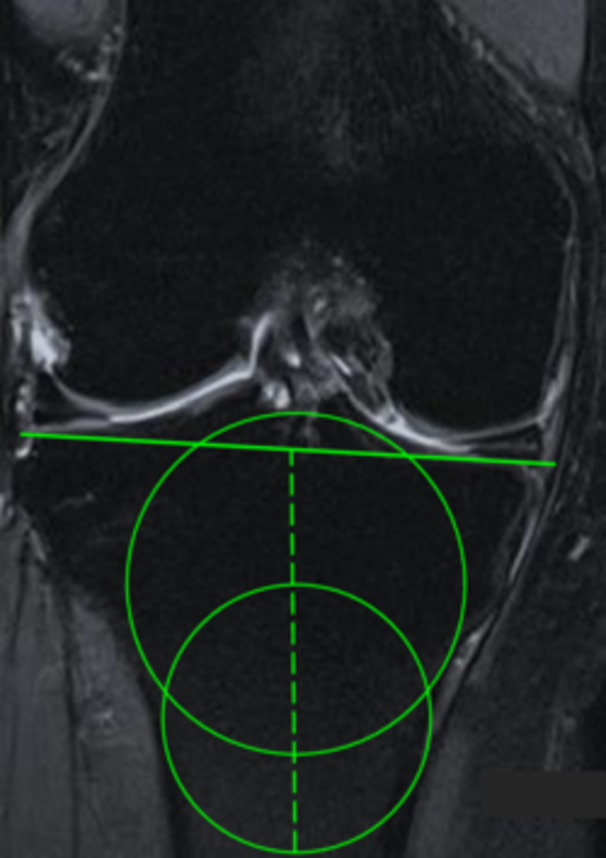
Magnetic resonance image of the medial proximal tibial angle. On the coronal plane in which the bilateral tibial eminences are clearly visualized, two circles are drawn, each tangent to the cortical margins of the bone. The line connecting the centres of these circles is defined as the tibial axis (long dashed line). The medial proximal tibial angle is measured as the medial angle between the tibial axis and the solid line connecting the most superior medial and lateral cortical edges.

On preoperative MRI, PSCL (present/absent) and other established MMPRT‐associated signs (cleft, giraffe neck and ghost signs) were recorded (Figure 1) [2, 5]. PSCL was defined as a focal high‐signal‐intensity lesion at the posterior tibial corner adjacent to the medial meniscus posterior root attachment on fluid‐sensitive sequences. Spontaneous osteonecrosis of the knee (SONK)/subchondral insufficiency fracture of the knee (SIFK) was recorded as a separate MRI finding and was defined as a broader subchondral lesion with a subchondral low‐signal‐intensity line and/or surrounding bone marrow oedema‐like signal in the medial femoral condyle or medial tibial plateau. When signal change was confined to the focal posterior tibial corner region typical of PSCL, without a subchondral low‐signal‐intensity line or broader subchondral involvement, it was classified as PSCL rather than SONK/SIFK. MME was measured, and a threshold of >3 mm was used to define severe extrusion [9]. PTS of the medial tibia plateau and MPTA were measured on sagittal (Figure 2) and coronal (Figure 3) MR images, respectively [7, 8, 12, 14]. MRI evaluations were performed for all included knees using short inversion time inversion recovery imaging. Measurements were performed by a single experienced orthopaedic surgeon who was blinded to the clinical data and surgical findings. For intraobserver and interobserver reliability assessments, measurements were repeated after a 2‐week interval in a randomly selected subset of 20 knees by two independent orthopaedic surgeons, and reliability was evaluated using the intraclass correlation coefficient (ICC). The ICC values indicated excellent reliability (intraobserver: PTS, 0.98; PSCL, 0.93; MME > 3 mm, 0.91; interobserver: PTS, 0.97; PSCL, 0.81; MME > 3 mm, 0.81).

### Statistical analyses

Continuous variables are presented as mean ± standard deviation and compared using Student's *t* test. Categorical variables are presented as counts (percentages) and were compared using Fisher's exact test. For comparisons among the three MRI timing categories (acute, subacute and chronic), proportions were assessed using the Cochran–Armitage trend test. Multivariable logistic regression was performed with PSCL positivity as the dependent variable and PTS as the primary independent variable, adjusting for MRI timing category. MRI timing category was entered as a categorical variable with the acute group as the reference. Odds ratios (ORs) with 95% confidence intervals (CIs) were calculated. Given the limited number of events, the number of variables included in the multivariable model was restricted to minimize the risk of overfitting. Model performance was assessed using Nagelkerke's *R* ^2^. Statistical significance was set at *p* < 0.05. Statistical analysis was performed using R version 4.0.3 (R Foundation for Statistical Computing).

## RESULTS

The analysis included 53 knees in 46 patients. The mean age and body mass index (BMI) of the patients were 66.5 ± 9.4 years and 25.3 ± 4.0 kg/m^2^, respectively. The overall PSCL prevalence was 58.5% (*n* = 31). Based on the time from onset to MRI, 22 knees were classified as acute (within 3 weeks), 16 as subacute (within 8 weeks but beyond 3 weeks) and 15 as chronic (beyond 8 weeks). PSCL prevalence decreased as the time from onset to MRI increased (acute, 86.4%, *n* = 19; subacute, 62.5%, *n* = 10; chronic, 13.3%, *n* = 2; *p* < 0.001) (Table 1).

**Table 1:** Differences in MRI‐derived outcomes by MRI timing category.

Finding	Acute (*n* = 22)	Subacute (*n* = 16)	Chronic (*n* = 15)	*p* for trend
PSCL	19 (86.4)	10 (62.5)	2 (13.3)	<0.001
Cleft sign	15 (68.2)	15 (93.8)	15 (100)	0.006
Giraffe neck	15 (68.2)	14 (87.5)	14 (93.3)	0.046
Ghost sign	15 (68.2)	15 (93.8)	15 (100)	0.006
MME > 3 mm	2 (9.1)	12 (75.0)	15 (100)	<0.001
SONK/SIFK	3 (13.6)	7 (43.8)	9 (60.0)	0.003

*Note*: Data are presented as *n* (%). Acute, within 3 weeks; subacute, within 8 weeks but beyond 3 weeks; chronic, beyond 8 weeks.

Abbreviations: MME, medial meniscal extrusion; MRI, magnetic resonance imaging; PSCL, posterior shiny‐corner lesion; SONK/SIFK, spontaneous osteonecrosis of the knee/subchondral insufficiency fracture of the knee.

In unadjusted comparisons, PSCL‐positive patients had higher PTS than PSCL‐negative patients (8.7° ± 3.4° vs. 5.5° ± 2.9°; *p* < 0.001) (Table 2). In the multivariable logistic regression adjusted for time category, a higher PTS was independently associated with PSCL positivity (OR, 1.35 per 1°; 95% CI, 1.05–1.74; *p* = 0.021) (Table 3). Compared with the acute MRI timing category, the chronic category was independently associated with lower odds of PSCL positivity (OR, 0.03; 95% CI, 0.004–0.26; *p* = 0.001). The multivariable logistic regression model demonstrated acceptable fit (Nagelkerke's *R* ^2 ^= 0.56). The full model specification, including coefficients and intercept, is provided in Data S1.

**Table 2:** Patient characteristics by PSCL status.

Variable	PSCL+ (*n* = 31)	PSCL− (*n* = 22)	*p* value
Age (years)	63.7 ± 9.9	68.3 ± 6.9	0.062
Female sex	27 (87.1)	14 (63.6)	0.054
BMI (kg/m^2^)	25.3 ± 4.3	24.9 ± 3.5	0.730
PTS (°)	8.7 ± 3.4	5.5 ± 2.9	<0.001
MPTA (°)	86.8 ± 1.4	86.9 ± 1.6	0.870
MME > 3 mm	11 (35.5)	18 (81.8)	0.002
SONK/SIFK	8 (25.8)	11 (50.0)	0.088
MRI timing (acute/subacute/chronic)	19/10/2	3/6/13	<0.001

*Note*: Data are presented as mean ± standard deviation, *n* (%) or *n*/*n*/*n*.

Abbreviations: BMI, body mass index; MME, medial meniscus extrusion; MPTA, medial proximal tibia angle; MRI, magnetic resonance imaging; PSCL, posterior shiny‐corner lesion; PTS, posterior tibial slope; SONK/SIFK, spontaneous osteonecrosis of the knee/subchondral insufficiency fracture of the knee.

**Table 3:** Multivariable logistic regression for PSCL positivity.

Predictor	Odds ratio	95% CI	*p* value
PTS (per 1° increase)	1.35	1.05–1.74	0.021
MRI timing: subacute versus acute	0.25	0.04–1.38	0.111
MRI timing: chronic versus acute	0.03	0.004–0.26	0.001

Abbreviations: CI, confidence interval; MRI, magnetic resonance imaging; PSCL, posterior shiny‐corner lesion; PTS, posterior tibial slope.

## DISCUSSION

This study investigated whether the PTS modifies the presence of PSCL in surgically treated MMPRT across different MRI timing categories. The most important finding of the present study is that PSCL appearance is not only determined by time from symptom onset but is also significantly influenced by PTS. MMPRT is a distinct clinical entity characterized by sudden loss of meniscal hoop tension and rapid progression of medial compartment degeneration [6]. The timing of MMPRT onset is a critical factor that directly influences the choice of surgical strategy [4, 11]. In clinical practice, the timing of MMPRT onset is often unclear, and imaging findings are frequently used to estimate whether a lesion is acute or chronic. Therefore, reliable imaging markers that reflect the lesion chronicity are important for clinical interpretation. Our results demonstrated that PSCL prevalence decreased progressively with time, whereas MME > 3 mm increased with chronicity. These findings support the concept that PSCL and MME represent different temporal aspects of MMPRT. PSCL may reflect an early subchondral bone marrow reaction related to acute loading at the root attachment, whereas MME likely represents a later structural consequence of meniscal dysfunction.

The lesions traditionally described as SONK are now often interpreted within the concept of SIFK [16]. In the present study, SONK/SIFK was evaluated separately from PSCL. This distinction is important because PSCL represents a focal posterior tibial corner signal adjacent to the medial meniscus posterior root attachment, whereas SONK/SIFK represents a broader subchondral lesion that may occur in the medial femoral condyle or medial tibial plateau.

PSCL has been reported as an early MRI finding following MMPRT onset, with higher sensitivity when MRI is performed within 3 or 8 weeks [13]. Although our data supported this time dependence, the time from onset alone did not fully explain PSCL heterogeneity. In the present study, higher PTS was independently associated with PSCL positivity. Importantly, our findings suggest that the presence of PSCL is not solely a time‐dependent phenomenon. Even in the acute phase, PSCL may be absent in knees with a low PTS. These findings have important implications for the clinical interpretation of PSCL as a marker of lesion chronicity. In particular, PSCL‐negative findings do not necessarily exclude acute MMPRT, especially in patients with a low PTS. From a clinical perspective, PSCL should be interpreted in conjunction with other MRI findings, such as MME, rather than as a standalone indicator of acute injury. Although PSCL may reflect early subchondral response, MME appears to represent later‐stage disease progression. Because PSCL is often interpreted as an early imaging marker, PSCL‐negative findings may lead to underestimation of acute MMPRT when other signs are subtle. Our findings suggest that PSCL should be interpreted in the context of tibial morphology, particularly PTS. However, it is important to emphasize that the present study demonstrates an association between PTS and PSCL positivity and does not establish a causal relationship. The underlying biomechanical mechanisms remain speculative and should be interpreted with caution.

This study has several limitations. First, this was a retrospective, single‐centre study with a modest sample size, and the cohort consisted only of surgically treated MMPRT cases. Therefore, selection bias may have been introduced, and the findings may not be generalizable to all patients with MMPRT, including those treated nonoperatively. Second, the MRI timing categories were based on patient‐reported symptom onset or injury. In elderly patients with MMPRT, symptom onset may be gradual or unclear, and potential recall bias could have affected classification into MRI timing categories. Third, this was a cross‐sectional study rather than a longitudinal serial imaging study. Therefore, the observed differences in PSCL prevalence across MRI timing categories were inferred from comparisons between different patients, not from direct temporal changes within the same knees. Fourth, although MMPRT was confirmed intraoperatively in all cases, detailed tear morphology, including complete root avulsion and radial tear adjacent to the posterior root attachment, was not analysed separately. Tear morphology or proximity to the root attachment may have influenced PSCL appearance. Fifth, SONK/SIFK was evaluated as a separate MRI finding from PSCL; however, both findings involve subchondral signal changes, and distinguishing focal PSCL from broader SONK/SIFK‐related changes may be challenging in some cases. Finally, although the multivariable model was restricted to reduce the risk of overfitting, the modest sample size may have affected the stability of the estimated ORs. These limitations should be considered when interpreting the association between PTS and PSCL positivity.

## CONCLUSION

PSCL is associated with early phase of MMPRT; however, its presence is influenced by the PTS and is not solely time‐dependent. PSCL may be absent even in acute cases with low PTS and therefore should not be used alone to exclude acute MMPRT.

## AUTHOR CONTRIBUTIONS

Ryo Sasaki, Kazuya Kaneda and Masaki Nagashima conceptualized and designed the study. Ryo Sasaki, Taichi Nishimura, Teppei Hayashi and Kazuya Kaneda performed the surgery. Ryo Sasaki and Kazuya Kaneda performed the experiments, analysed the data and drafted the manuscript. Hideo Morioka organized the research team. All authors edited and approved the manuscript prior to submission.

## CONFLICT OF INTEREST STATEMENT

The authors declare no conflicts of interest.

## ETHICS STATEMENT

This study was approved by the institutional review board of NHO Tokyo Medical Center (IRB no. R25‐049). The requirement for informed consent was waived due to the retrospective nature of the study.

## Supporting information

Supporting Table.

Supporting File.

## Data Availability

The datasets used in this study are available from the corresponding author upon request.
