# Supplementary material for: The posterior tibial slope modifies the diagnostic utility of posterior shiny‐corner lesions in medial meniscus posterior root tears
Source: J Exp Orthop. 2026 Jul 9;13(3):e70843. doi: 10.1002/jeo2.70843 (PMC13349080; doi:10.1002/jeo2.70843)
Supplement: Supplementary file 1 — Supporting Table. [file JEO2-13-e70843-s002.docx]

**Online Resource 1.** Full specification of the multivariable logistic regression model for PSCL positivity

| Predictor | β coefficient | Odds ratio | 95% CI | P value |
| --- | --- | --- | --- | --- |
| Intercept | −0.296 | − | − | 0.775 |
| PTS (per 1° increase) | 0.301 | 1.35 | 1.05–1.74 | 0.021 |
| MRI timing: acute vs subacute | −1.401 | 0.25 | 0.04–1.38 | 0.111 |
| MRI timing: acute vs chronic | −3.388 | 0.03 | 0.004–0.26 | 0.001 |

Model performance: Nagelkerke’s R² = 0.56

MRI timing was entered as a categorical variable with the acute group as the reference.

PSCL, posterior shiny-corner lesion; PTS, posterior tibial slope; MRI, magnetic resonance imaging.
