# Supplementary material for: The posterior tibial slope modifies the diagnostic utility of posterior shiny‐corner lesions in medial meniscus posterior root tears
Source: J Exp Orthop. 2026 Jul 9;13(3):e70843. doi: 10.1002/jeo2.70843 (PMC13349080; doi:10.1002/jeo2.70843)
Supplement: Supplementary file 2 — Supporting File. [file JEO2-13-e70843-s001.docx]

**STROBE Checklist for Observational Studies (Cohort Study)**

Manuscript title: The Posterior Tibial Slope Modifies the Diagnostic Utility of Posterior Shiny-Corner Lesions in Medial Meniscus Posterior Root Tears

| Item No. | Recommendation | Reported (Page/Section) |
| --- | --- | --- |
| 1(a) | Indicate study design in title/abstract | Title/Page 2, Abstract |
| 1(b) | Provide balanced summary | Pages 2-3, Abstract |
| 2 | Background/rationale | Page 4, Introduction |
| 3 | Objectives | Pages 4-5, Introduction (last paragraph) |
| 4 | Study design | Page 5, Methods (Study design) |
| 5 | Setting | Page 5, Methods (Study design and participants) |
| 6 | Participants | Page 5, Methods (Inclusion criteria) |
| 7 | Variables | Pages 5-6, Methods (Definitions of PSCL, PTS, MME) |
| 8 | Data sources/measurement | Pages 5-6, Methods (MRI evaluation) |
| 9 | Bias | Page 9, Discussion (limitations) |
| 10 | Study size | Page 5, Methods |
| 11 | Quantitative variables | Page 6, Methods |
| 12 | Statistical methods | Page 6, Methods |
| 13(a) | Participants (numbers) | Page 7, Results |
| 13(b) | Reasons for non-participation | Not applicable |
| 13(c) | Flow diagram | Not included |
| 14(a) | Descriptive data | Results (Table 1) |
| 14(b) | Missing data | Not explicitly stated |
| 15 | Outcome data | Page 7, Results |
| 16(a) | Main results | Results (Tables 2 and 3) |
| 16(b) | Category boundaries | Pages 5-6, Methods |
| 16(c) | Absolute risk | Not applicable |
| 17 | Other analyses | Not applicable |
| 18 | Key results | Pages 7-8, Discussion |
| 19 | Limitations | Page 9, Discussion |
| 20 | Interpretation | Pages 8-9, Discussion |
| 21 | Generalizability | Page 9, Discussion |
| 22 | Funding | Not applicable |
